# EZmito2: a tool suite for mitochondrial genome dataset preparation, population genetics, and visualization

**DOI:** 10.1093/molbev/msag210

**Published:** 2026-08-19

**Authors:** Claudio Cucini, Joan Pons, Rebecca Funari, Antonio Carapelli, Francesco Frati, Francesco Nardi

**Affiliations:** Department of Life Sciences, University of Siena, Siena 53100, Italy; National Biodiversity Future Center (NBFC), Palermo 90133, Italy; Department of Animal and Microbial Biodiversity, Mediterranean Institute for Advanced Studies (IMEDEA), Esporles 07190, Spain; Department of Life Sciences, University of Siena, Siena 53100, Italy; Department of Life Sciences, University of Siena, Siena 53100, Italy; National Biodiversity Future Center (NBFC), Palermo 90133, Italy; Interuniversity Center for Studies on Bioinspired Agro-Environmental Technology (BAT Center), Naples 80055, Italy; Department of Life Sciences, University of Siena, Siena 53100, Italy; National Biodiversity Future Center (NBFC), Palermo 90133, Italy; Interuniversity Center for Studies on Bioinspired Agro-Environmental Technology (BAT Center), Naples 80055, Italy; Department of Life Sciences, University of Siena, Siena 53100, Italy; National Biodiversity Future Center (NBFC), Palermo 90133, Italy; Interuniversity Center for Studies on Bioinspired Agro-Environmental Technology (BAT Center), Naples 80055, Italy

**Keywords:** web server, mitogenome, nucleotide bias, relative synonym codon usage, mitochondrial map, genetic distances

## Abstract

Mitogenomic datasets are central to molecular evolution and phylogenetics, yet preparatory workflows remain labor-intensive and prone to errors introduced during manual data preparation. EZmito2 is a re-implementation of the widely used EZmito pipeline that offers a fully reproducible and user-accessible solution for mitogenomic dataset curation and result visualization. It is accessible via a public web server for rapid analyses and through local installation, enabling reproducible workflows on personal computers or computational clusters. The pipeline consolidates the core modules—EZpipe, EZskew, and EZcodon—and extends functionality through newly developed tools for genome visualization (EZcircular, EZmap), chimeric region detection (EZmix), gene extraction from NCBI-deposited genomes (EZsplit), structural annotation of transmembrane domains in mitochondrial protein-coding genes (EZtrampo), and population genetic studies (EZdist, EZpcoa, EZpopstat). All tools accept standard input formats and generate ready-to-publish outputs. By providing a user-friendly platform for mitogenomic exploration and quality control, EZmito2 facilitates reproducible analyses for evolutionary and molecular research communities.

## Graphical Abstract

**Graphical Abstract:**
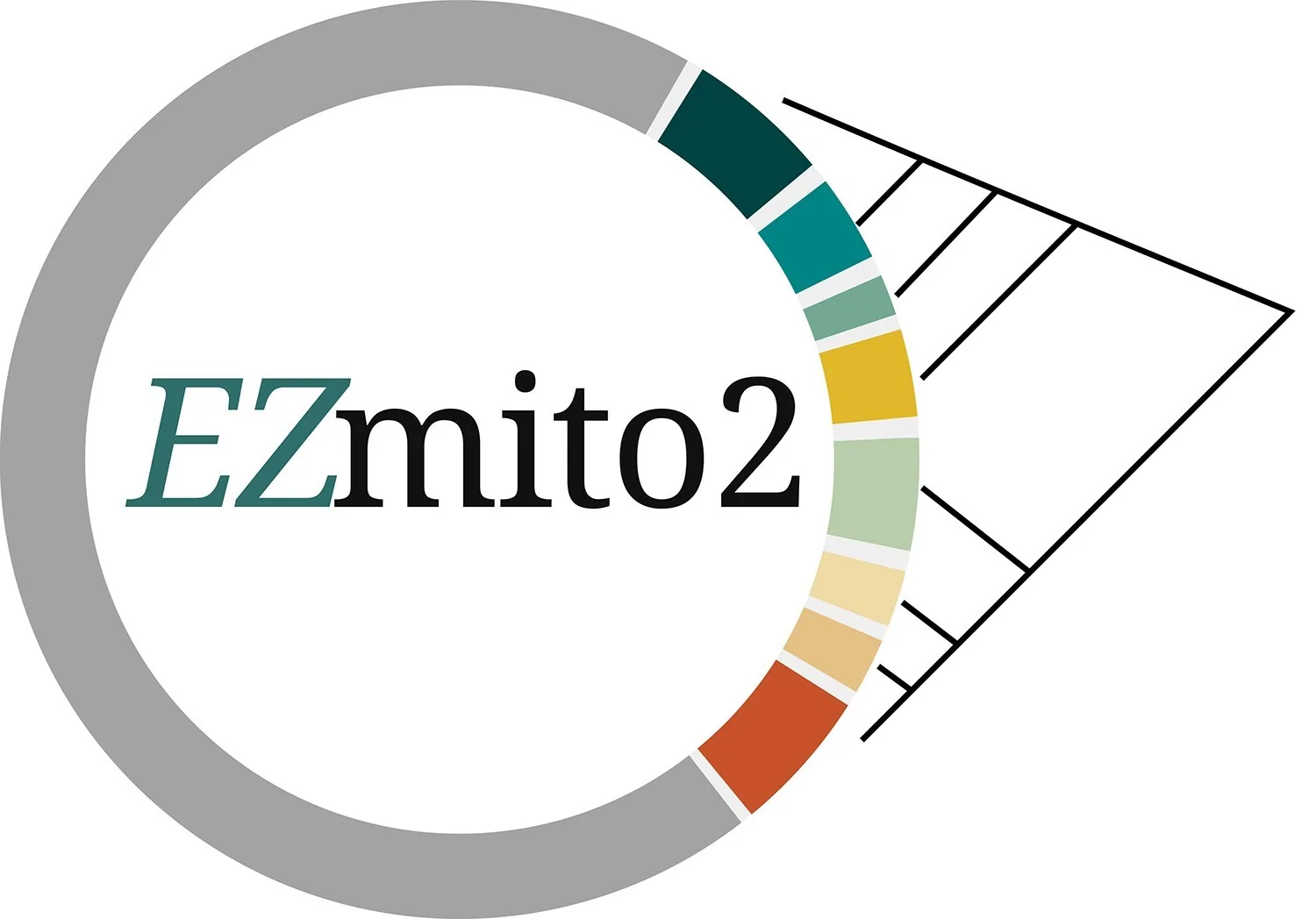

## Introduction

In the genomic era, mitochondrial genomes remained a valuable marker for phylogenetic and phylogeographic studies. Their high copy number per cell, predominantly uniparental inheritance, and alternation of regions characterized by different substitution rates compared to nuclear genes, combined with the ease of assembling them from low-coverage sequencing data, make mitochondrial genomes especially useful when nuclear data are less practical to obtain. This includes cases such as fossils, museum specimens, eDNA, and large-scale taxon surveys, and also facilitates insights into understudied taxa (Blair 2023; Cameron 2025).

The rapid accumulation of complete mitochondrial genome sequences has shifted the bottleneck in mitogenomic research from data generation to dataset preparation, validation, and reproducible analysis. This trend has been driven by decreasing sequencing costs and the feasibility of mitogenome recovery from diverse data sources, including low-coverage whole-genome sequencing (Therkildsen and Palumbi 2017), metagenomic (Baeza et al. 2026), RAD-seq (Stobie et al. 2019), and RNA-seq data (Forni et al. 2019).

While numerous tools exist for mitogenomic research, covering miotochondrial assembly and annotation (e.g. NOVOplasty; Dierckxsens et al. 2017; MitoZ; Meng et al. 2019; and MITOS2; Bernt et al. 2013), sequence alignment and phylogenetic inference (e.g. NGPhylogeny; Lemoine et al. 2019; MitoPhast; Tan et al. 2015; or PhyloSuite; Zhang et al. 2020) and population genetics statistics (e.g. DNAsp; Rozas et al. 2017; and Arlequin; Excoffier et al. 1992), routine preparatory steps and result visualization remain labor-intensive and prone to user error. These include manual dataset curation, gene extraction, annotation standardization, sequence orientation checks, and file-format conversions between software tools. At a broader scale, tools such as PHLAWD (Smith et al. 2009), PhylotaR (Bennett et al. 2018), and SuperCRUNCH (Portik and Wiens 2020) have been developed to automate large-scale phylogenetic dataset assembly from public databases, but their scope targets supermatrix construction across diverse loci rather than mitogenome-specific dataset preparation, which remains a persistent bottleneck.

**Figure 1:**
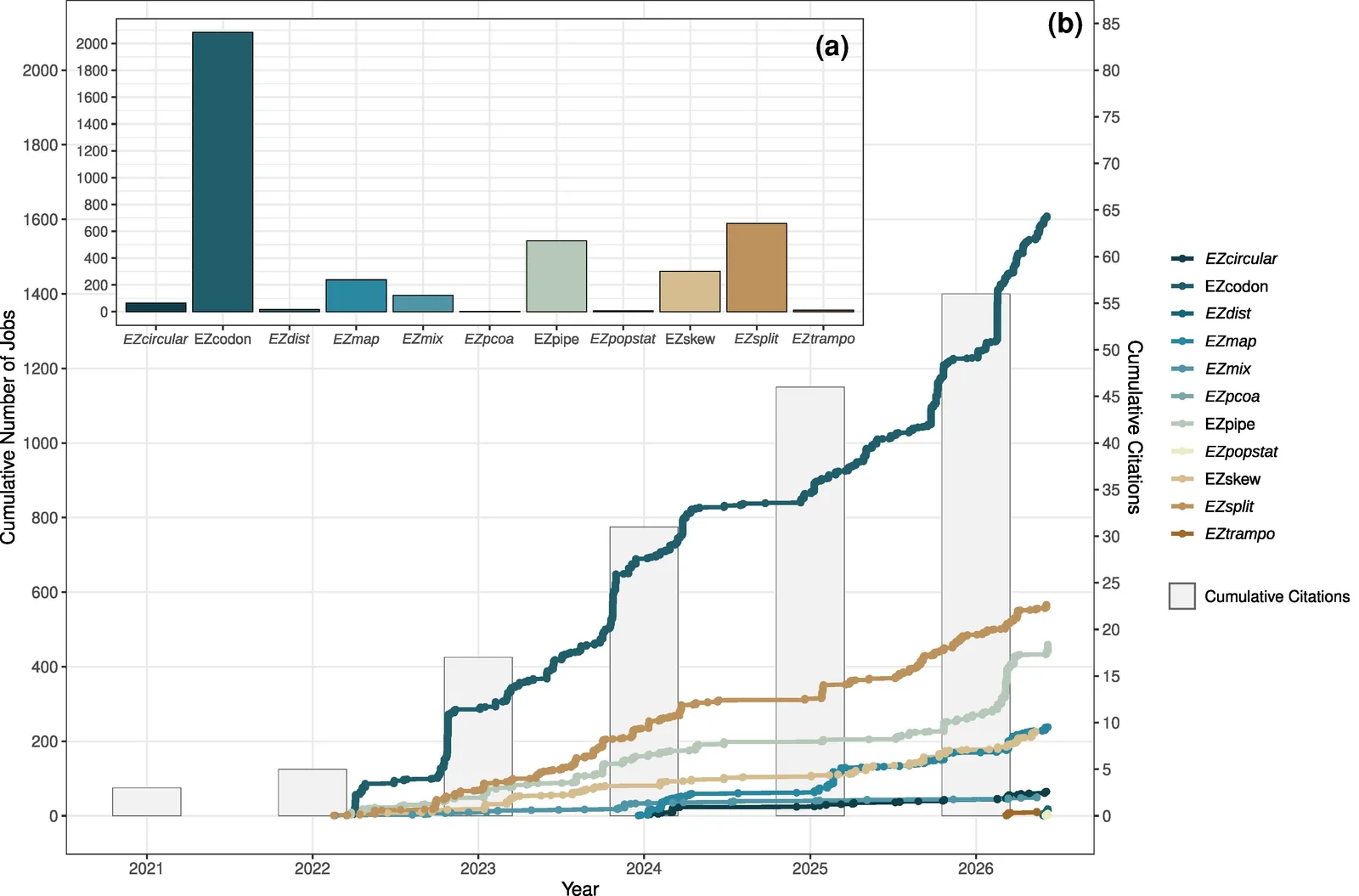
Usage statistics of EZmito since its introduction. a) Total number of jobs submitted to each EZmito tool. b) Temporal trends in EZmito usage. Points indicate the cumulative number of submitted jobs (from 2022 onward, the year in which job submissions began to be systematically recorded), whereas bars represent the cumulative number of citations indexed in Scopus. Data were collected in June 2026. Tools are color-coded; names shown in italics correspond to modules introduced after the original release of EZmito.

In this context, EZmito (Cucini et al. 2021) was initially developed to automate the preparation of mitochondrial protein-coding gene (PCGs) datasets, providing a guided workflow to uniform analyses across taxa. The growing adoption of EZmito by the research community (Fig. 1) demonstrates its utility, but the original multi-language architecture (Python, R, Bash) limited maintainability, portability, and ease of local installation. Moreover, since its initial release, the original toolbox has grown in terms of available software, some of which have never been formally described and presented to the scientific community (Fig. 1).

Here we present EZmito2, a fully Python-based reimplementation that preserves the original analytical logic while improving reproducibility, execution speed, and usability. It is accessible via a web interface for rapid and interactive use, as well as through a local Conda-based installation for integration into custom workflows, providing a standardized and robust solution for mitogenomic research and haplotype-based studies.

## Software architecture and availability

EZmito2 is a complete rewrite of the original EZmito software (Cucini et al. 2021), consolidating the previously heterogeneous multi-language codebase (Python, R, Bash) into a single, self-contained Python code. EZmito2 is accessible via two deployment modes: a publicly available web server (http://ezmito.unisi.it), hosted on a virtual machine at the University of Siena, providing a browser-based interface requiring no installation; and a local Conda installation, recommended for integration into automated workflows, high-throughput analyses, and computationally intensive jobs, which benefit from dedicated computational resources. Both versions share identical analytical logic and produce equivalent outputs. Graphics are generated using matplotlib v3.10.8 (Hunter 2007), with the exception of EZtrampo, which additionally employs plotly v6.5.0 (Plotly Technologies Inc. 2015) to maintain compatibility with its original design (Cucini et al. 2026). All graphical outputs are rendered as vector formats (SVG, PDF), ensuring suitability for direct inclusion in publications. Each module incorporates a sanity check that screens input file validity and detects common formatting errors. Any input failing these validations is flagged in the run log and in a structured error-report file that identifies the step at which the failure occurred, provides a plain-language description of the cause, and suggests corrective actions, improving usability and accessibility for researchers without specialist bioinformatics training.

**Figure 2:**
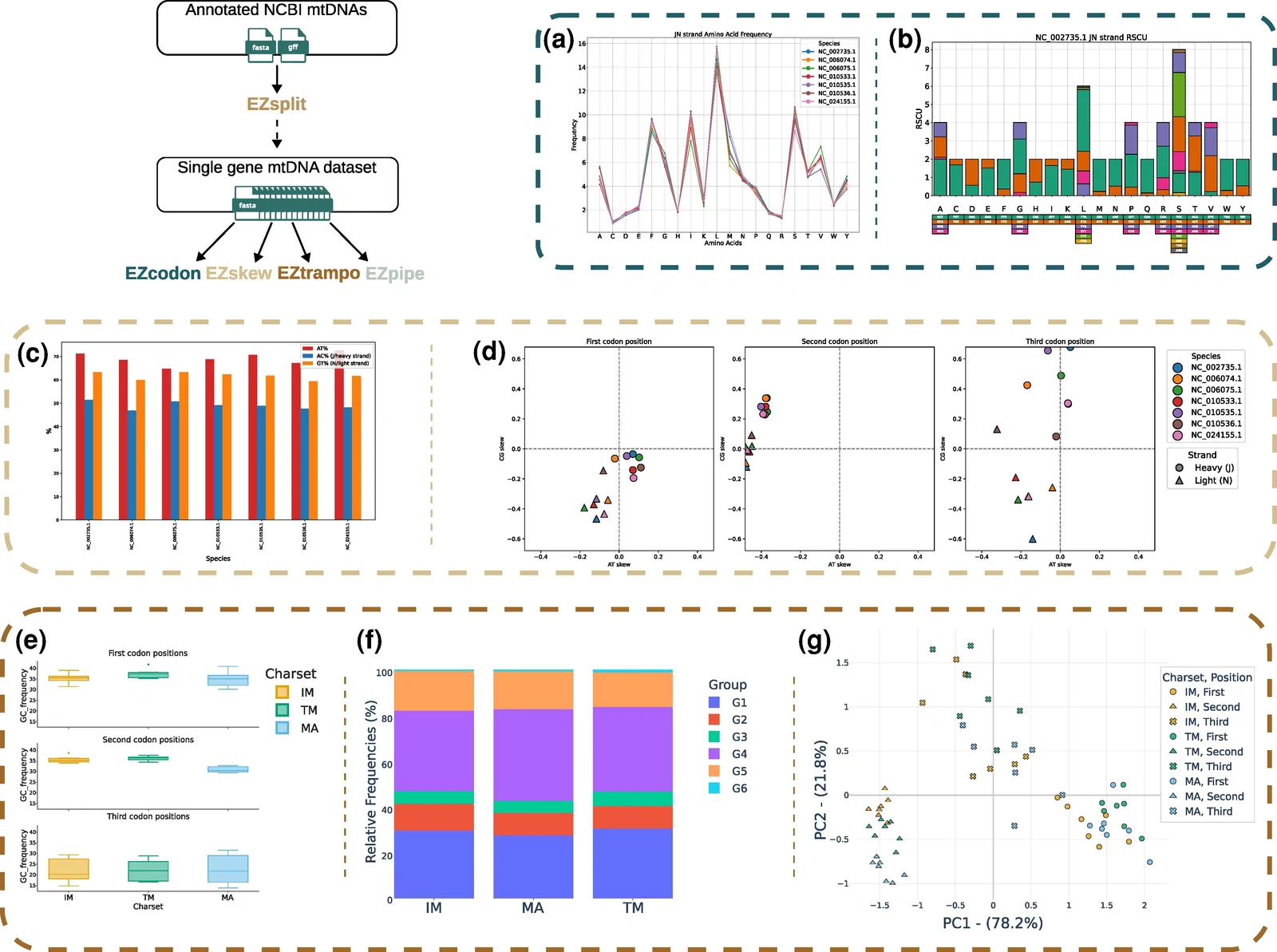
Overview of mitochondrial dataset preparation and nucleotide composition analysis. A possible workflow is represented by the download of annotated mitogenomes from NCBI for protein-coding gene extraction using EZsplit, followed by nucleotide compositional analysis with EZcodon and EZskew, or dataset preparation for phylogenetic inference through EZpipe and EZtrampo. Input file requirements are depicted with file format labels for each tool. Graphical outputs of each tool are shown in (a–g) and color-coded by tool. a) EZcodon amino acid frequency plot color-coded by species; b) Relative Synonymous Codon Usage, plotted per species and color-coded by codon; c) EZskew genome-level compositional biases: A + T% computed mitogenome-wide, and A + C% and G + T% computed on the heavy (J) and light (N) strands respectively, color-coded by metric; d) strand- and codon-position-specific skews: AT and CG skews computed separately for the J and N strands across all three codon positions, with species color-coded and strands distinguished by shape. EZtrampo secondary structure-based partitioning: e) GC content frequency by structural domain defined as inter-membrane (IM), mitochondrial matrix (MA) and transmembrane (TM) across all analyzed species; f) amino acid frequencies grouped into six physicochemical classes across transmembrane domains (IM, MA, TM); g) AT and CG skew principal component analysis (PCA) plot by codon position and membrane domain. Datasets from Cucini et al. (2026).

**Figure 3:**
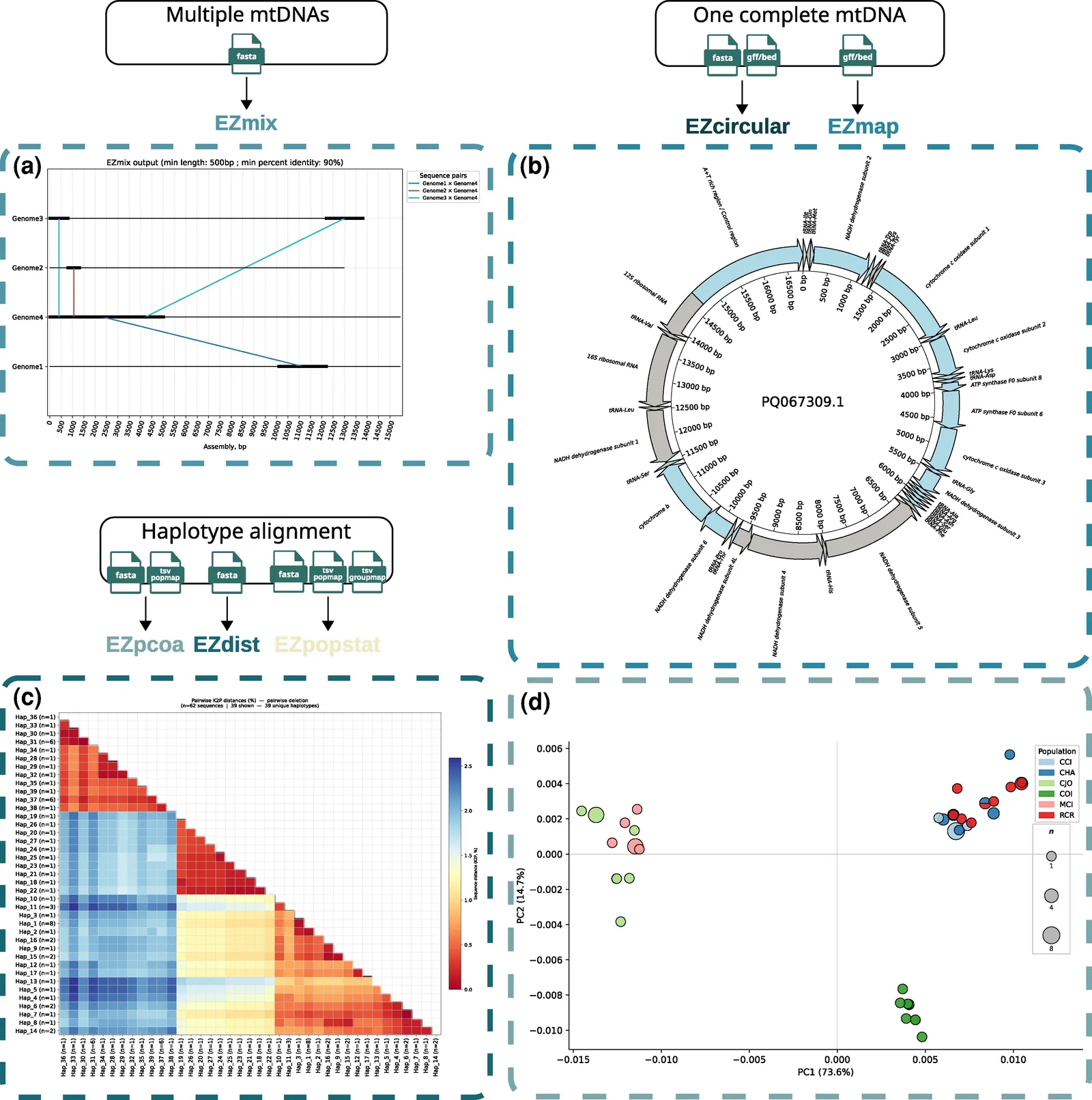
Overview of chimeric assembly detection, genome visualization and population-level analyses. Three possible workflows are represented: the screening of multiple mitogenomes for chimeric assemblies and their visualization using EZmix, EZcircular, and EZmap, as well as the population-level analysis of aligned haplotype sequences through EZdist, EZpcoa, and EZpopstat. Input file requirements are depicted with file format labels for each tool. a) EZmix pairwise sequence similarity output across four input genomes; colored lines indicate BLAST hits above the default threshold of minimum length (500 bp) and percent identity (90%), with the underlying assemblies rendered as black blocks; the absence of cross-genome signal confirms assembly integrity. b) EZmap circular map of a complete mitochondrial genome with color-coded gene-strand annotations. c) EZdist triangular heatmap of pairwise Kimura two-parameter (K2P) distances (%) computed with pairwise deletion across unique haplotypes. d) EZpcoa ordination plot in which each data point is rendered as a pie chart with slices proportional to haplotype frequency within each population, color-coded by population. Datasets from Nardi et al. (2024), Boschi et al. (2025) and Cucini et al. (2026).

EZmito2 integrates and extends the original EZmito pipeline, entailing an update of the original three tools and the introduction of eight new modules (Fig. 1). Although each tool can be used independently, we discuss them within three common analytical workflows (Figs 2 and 3). The first group covers dataset preparation, nucleotide composition analysis, and preparatory steps for phylogenetic inference, and includes EZsplit, EZcodon, EZskew, EZpipe, and EZtrampo. The second group provides genome visualization and chimeric assembly detection, encompassing EZcircular, EZmap, and EZmix. The third group extends the original platform to population-level analyses through EZdist, EZpcoa, and EZpopstat. All modules accept standard file formats (FASTA, GFF, BED, TSV) and produce output files ready for downstream use or direct publication. A detailed description of parameters, input requirements, and output formats for each module is provided in File S1, in the software repository (https://github.com/ESZlab/EZmito2) and on the web server application pages (http://ezmito.unisi.it/).

## Workflow and applications

The eleven EZmito2 modules address distinct stages of mitogenomic data preparation and analysis. However, since some tools' inputs and outputs can be employed for multiple tools and/or downstream analyses, we discuss three possible common workflows.

### Dataset preparation and nucleotide composition analysis

A common starting point for mito-phylogenomics is a set of annotated mitogenomes downloaded from NCBI as multi-genome FASTA files accompanied by GFF annotations. EZsplit extracts individual protein-coding genes based on annotated boundaries, producing per-gene multi-taxon FASTA files with standardized headers ready for downstream analysis. The EZsplit output can serve as primary input for four complementary analytical modules: EZcodon, EZskew, EZpipe (the core tools from the original version of EZmito), and EZtrampo (Fig. 2). See File S1 for a detailed description and usage guidelines of the tools. EZcodon computes amino acid frequencies (Fig. 2a) and Relative Synonymous Codon Usage (RSCU) using the cai2 package (Fig. 2b; Lee 2018) following translation and strand-aware concatenation of PCGs. EZskew computes nucleotide compositional biases across strands and codon positions. A + T content, being strand-independent (Formaggioni et al. 2021), is calculated over the full concatenated PCG matrix. In turn A + C and G + T contents, which are strand-dependent (Hassanin et al. 2005), are computed separately over heavy- (J) and light-strand (N) concatenations (Fig. 2c). AT and CG skews are then computed per codon position for each strand following Hassanin et al. (2005) (Fig. 2d). Both EZskew and EZcodon accept the same input format, namely a set of per-gene FASTA files (assigned to either the J or the N strand) and the corresponding NCBI genetic code. Unlike the original implementation, strand-independent analyses are supported, allowing datasets containing only J- or only N-oriented genes to be analyzed without modification.

EZpipe prepares PCG datasets for phylogenetic inference: sequences are translated into amino acids following the genetic code selected by the user, aligned with MAFFT (Katoh and Standley 2013), retro-aligned to preserve the reading frame, trimmed of hypervariable regions with PyGblocks (Castresana 2000, as implemented in https://github.com/iTaxoTools/pygblocks) under strict codon-aware parameters, and concatenated into PHYLIP format via biopython (Cock et al. 2009) (see File S1 for further information). Partition schemes compatible with PartitionFinder2 (Lanfear et al. 2017) are generated alongside the alignment, with partitions defined by gene and codon position. Third codon positions can be optionally excluded.

EZtrampo implements the TRAMPO pipeline (Cucini et al. 2026) to extend codon-position partitioning with protein secondary structure information. Several mitochondrial PCGs encode subunits of the oxidative phosphorylation complexes that are integral inner-membrane proteins, whose transmembrane (TM) helical segments experience markedly different selective pressures from the hydrophilic regions facing the mitochondrial matrix (MA) or the intermembrane space (IM). Ignoring this structural heterogeneity may lead to substitution model misspecification in phylogenetic analyses (Cucini et al. 2026). EZtrampo annotates each codon in the alignment with its domain assignment (TM, MA, or IM) based on a reference database built in TRAMPO v.2024.1 from UniProt-SwissProt reference sequences (UniProt Consortium 2025), with domain boundaries inferred by a set of predictive tools (TMHMM v2.0 (Krogh et al. 2001) HMMTOP v2.0 (Tusnády and Simon 2001) CCTOP v1.1.0 (Dobson et al. 2015)). EZtrampo accepts the same input requirements as EZpipe, namely a per-gene FASTA input and the NCBI genetic code employed for protein translation. The tool produces a suite of NEXUS partition files combining domain, codon position, gene, and optionally strand information (up to 117 partitions in the most detailed scheme) for direct use in PartitionFinder2, IQ-TREE3 (Wong et al. 2026), or Bayesian inference software such as MrBayes (Ronquist et al. 2012). Complementary summary tables and plots of nucleotide composition (Fig. 2e), domain-specific physicochemical properties (Fig. 2f), and skews (Fig. 2g) are produced.

### Chimeric assembly detection and genome visualization

EZmix identifies candidate chimeric assemblies in multi-genome FASTA datasets, a recognized source of error in mito-metagenomic studies where related taxa co-sequenced as non-barcoded libraries may produce chimeric assemblies (Cucini et al. 2020). Pairwise BLASTn (Camacho et al. 2009) comparisons are performed across input genomes, and hits exceeding a user-defined threshold of percent identity and minimum alignment length are retained. The output plot displays the presence of highly similar regions, hypothetically derived from a misassembly, across input sequences (if present) through colored lines (Fig. 3a). The absence of shared sequences is itself an informative null result confirming assembly integrity.

EZcircular and EZmap provide complementary functions for working with the complete mitochondrial genome. EZcircular reorients the genome relative to a user-specified starting gene prior to rendering. Meanwhile, EZmap produces color-coded linear or circular genome feature maps distinguishing CDS, tRNA, rRNA, and control region annotations with separate colors for each coding strand (Fig. 3b). Both modules accept GFF or BED format.

### Population-level analyses

EZmito2 extends the analysis level from purely phylogenetic dataset preparation/visualization to population-level and phylogeographic studies with three additional tools. In this view, aligned sequence FASTA files are the primary input, optionally accompanied by a population map (required for EZpcoa) or group map files in TSV format that assign sequences to populations or user-defined groups. EZdist computes pairwise genetic distances between all sequences using uncorrected p-distances or Kimura two-parameter (K2P; Kimura 1980) distances, with the possibility of pairwise or complete gap deletion. Results are returned as a triangular distance matrix in both tabular and heatmap format (Fig. 3c). EZpcoa performs dimensionality reduction on haplotype frequency data using Principal Coordinates Analysis (PCoA) with user control over the number of components retained. Each data point is rendered as a pie chart in which slices are proportional to the frequency of the haplotype in each population, allowing simultaneous visualization of genetic distances between haplotypes and their distribution among populations (Fig. 3d). EZpopstat computes standard population genetic summary statistics such as haplotype diversity (*H*), nucleotide diversity (*π*), segregating sites (*S*), Tajima's D (Tajima 1989) and $F_{ST}$ (Hudson 1992; Tables S1, S2), providing a concise summary of genetic variation in a given set of sequences. Moreover, EZpopstat computes the Analysis of Molecular Variance (AMOVA; Excoffier et al. 1992), a permutation-based method that partitions total genetic variance into within- and among-group components, providing a measure of population differentiation analogous to *F*-statistics (Table S3). Group-level comparisons are available when a group map file is provided.

## Performance benchmarking

We benchmarked EZmito2 tools using datasets from our recent works (Nardi et al. 2024; Boschi et al. 2025; Cucini et al. 2026), tested on both the web server and a local installation. The web server is hosted on a virtual machine at the University of Siena and runs on a single AMD EPYC 7301 processor with 3.8 GB RAM under Debian GNU/Linux 12 (Bookworm), while local tests were performed on an Intel Core i7-14700K processor with 32 GB RAM running Ubuntu 24.04.4 LTS. Compared to the original multi-language EZmito web server (Cucini et al. 2021), EZmito2 demonstrates markedly faster processing across nearly all modules, with local execution generally outperforming web server execution. Processing times for core tools decreased substantially: EZcodon ran up to 14-fold faster, EZpipe up to 44-fold faster, and EZskew up to 4-fold faster compared to the original implementation (Table S4, Cucini et al. 2021). The newly introduced EZtrampo module showed up to 1.7-fold faster processing compared to the original TRAMPO script, across both dataset sizes and platforms (Table S2, Cucini et al. 2026). The other new tools (EZmix, Ezmap, Ezcircular, EZsplit, EZdist, EZpcoa, EZpopstat) runtimes varied depending on both the size of the analyzed dataset and the platform employed (Table S4).

## Discussion

EZmito2 marks a substantial update to the original EZmito pipeline (Cucini et al. 2021), motivated by two complementary goals: improving the technical robustness of the existing tools and expanding the analytical scope of the platform. By consolidating all modules into a single Python code, EZmito2 addresses the primary limitations of its original implementation, primarily related to a multi-language architecture (Python, R, Bash) that complicated maintenance, reproducibility, as well as the possibility of installing the package locally. The adoption of a Conda-based deployment model further lowers the barrier to local use, enabling integration into cluster workflows and reproducible research pipelines. The performance benchmarking confirms that the reimplementation produces substantial speed improvements across all core modules. Notably, local execution consistently outperforms web server execution, as expected given the dedicated hardware available on personal workstations. Together, these results confirm that EZmito2 is well-suited for medium- to large-scale analyses, even on modest local hardware.

Beyond performance, EZmito2 introduces eight new tools that collectively broaden the platform's utility across the mitogenomic research workflow. EZcircular and EZmap provide complementary genome visualization capabilities from BED and GFF annotation files, addressing a gap in the original pipeline, which produced no graphical genome representations. EZmix fills a specific niche in mito-metagenomic research, where possible chimeric assemblies arising from co-sequenced related taxa represent a recognized but often overlooked source of error (Cucini et al. 2020). EZsplit streamlines the extraction of individual PCGs from NCBI-deposited annotated genomes, a routine but time-consuming task that previously required manual curation or bespoke scripting. EZtrampo integrates the TRAMPO partitioning framework (Cucini et al. 2026) directly into the EZmito2 environment, enabling users to incorporate transmembrane domain information into phylogenetic partitioning schemes — an approach shown to improve model fitness and phylogenetic accuracy in mitochondrial datasets. Finally, a set of three new tools (EZdist, EZpcoa, EZpopstat) aimed at summarizing and visualizing population genetic statistics expands the possible application of the software.

Unlike general-purpose phylogenetic platforms such as PhyloSuite (Zhang et al. 2020), SuperCRUNCH (Portik and Wiens 2020), and NGPhylogeny (Lemoine et al. 2019), EZmito2 focuses on the preparatory and visualization steps that precede phylogenetic inference and does not include tree-building or model-selection steps. Its strength lies in providing a guided, standardized, and error-checked workflow for the specific stages that are most routinely performed in mitogenomic and population genetics research. The sanity-check procedures implemented across all input-accepting modules are particularly valuable in this regard, detecting common issues such as stop codons within sequences, frame-disrupting gaps, and inconsistent taxon labelling before they propagate to downstream analyses. EZmito2 does not integrate directly with genome assembly or annotation tools, and users must obtain annotated genomes from dedicated upstream software before applying the pipeline. We consider this design a strength as it leaves users free to choose the assembly and annotation tools best suited to their taxon and sequencing strategy without imposing a fixed upstream dependency. To maximize accessibility and facilitate adoption by non-specialist users, we provide this implementation as a user-friendly online platform that obviates the need for advanced bioinformatic expertize, thereby enabling broader and more effective utilization of the method.

Future development will focus on expanding the analytical modules — particularly in the area of visualization and compositional analysis — as well as on maintaining compatibility with evolving third-party dependencies such as MAFFT and BLASTn. Usage statistics from the web server (Fig. 1) will continue to guide prioritization of new features, as they have since the original release.

## Supplementary Material

msag210_Supplementary_Data

## Data Availability

The data and scripts underlying this article are available in the GitHub project (https://github.com/ESZlab/EZmito2) and on EZmito2's web server (http://ezmito.unisi.it/). No new sequences were generated in support of this research.
