# Supplementary material for: EZmito2: a tool suite for mitochondrial genome dataset preparation, population genetics, and visualization
Source: Mol Biol Evol. 2026 Aug 19;43(8):msag210. doi: 10.1093/molbev/msag210 (PMC13524090; doi:10.1093/molbev/msag210)
Supplement: Supplementary file 1 [file msag210_supplementary_data.zip › FileS1.pdf]

EZmito2

User Guide

A bioinformatic toolkit for mitogenomic analysis

Version 2026.6

https://ezmito2.unisi.it
https://github.com/ESZlab/EZmito2

Table of Contents

1. Introduction........ 4
1.1 Strand Convention........ 4
1.2 Mitochondrial Genetic Codes........ 5
2. General Input Requirements........ 6
2.1 FASTA Format........ 6
2.2 Per-gene FASTA Files........ 6
2.3 Annotation Formats........ 7
2.4 Population Map and Group Map........ 7
3. Tool Reference........ 9
3.1 EZsplit — Gene Extraction from Annotated Mitogenomes........ 9
3.2 EZcircular — Mitogenome Re-orientation........ 10
3.3 EZmap — Mitogenome Graphical Mapping........ 12
3.4 EZpipe — Phylogenetic-ready Nucleotide Matrix........ 13
3.5 EZcodon — Codon Usage Analysis........ 16
3.6 EZskew — Nucleotide Compositional Bias Analysis........ 18
3.7 EZmix — Inter-mitogenome Similarity Detection........ 20
3.8 EZtrampo — Transmembrane Domain Partition Analysis........ 22
3.9 EZpopstat — Population Genetics Statistics........ 27
3.10 EZpcoa — Ordination of Genetic Variation........ 30
3.11 EZdist — Ordination of Genetic Variation........ 32
4. Running EZmito2 Locally........ 35
4.1 EZsplit........ 35
4.2 EZcircular........ 35
4.3 EZmap........ 36
4.4 EZpipe........ 36
4.5 EZcodon........ 36
4.6 EZskew........ 37
4.7 EZmix........ 37
4.8 EZtrampo........ 37
4.9 EZpopstat........ 38
4.10 EZpcoa........ 38
4.11 EZdist........ 38
5. References........ 39

1. Introduction

EZmito2 is an integrated bioinformatic toolkit designed to streamline the preparation, analysis, and visualisation of mitochondrial genome (mitogenome) data for evolutionary and phylogenetic studies. The package encompasses eleven specialised tools that collectively address some of the analytical challenges encountered in mitogenomics: dataset assembly, sequence alignment, nucleotide compositional bias, codon usage, inter-molecular similarity screening, partition scheme generation, population genetics, and ordination.

This guide documents the theoretical basis, input requirements, statistical methods, and output interpretation for each tool. For software installation and command-line usage, see the companion **Installation Guide** available at https://github.com/ESZlab/EZmito2#-installation.

All tools are implemented in Python and rely on standard scientific packages: NumPy (Harris et al. 2020), pandas (McKinney 2010), Matplotlib (Hunter 2007), plotly (Plotly Inc. 2015) and SciPy (Virtanen et al. 2020). Sequence input/output and feature parsing are handled by Biopython (Cock et al. 2009). Library citations specific to individual tools are given in the corresponding **Method** subsections.

1.1 Strand Convention

The two strands of the mitochondrial genome are referred to throughout this guide using the following convention:

- **J strand** (*maJor* strand; also termed the Heavy strand or H-strand in the vertebrate literature): the strand that carries the majority of protein-coding genes.
- **N strand** (*miNority* strand; also termed the Light strand or L-strand): the complementary strand, encoding a minority of protein-coding genes in most metazoan mitogenomes.

EZmito2 requires the user to assign each gene to the correct strand when strand-aware analyses are requested (EZcodon, EZskew).

1.2 Mitochondrial Genetic Codes

The translation table used to verify codon integrity, detect internal stop codons, and translate nucleotide sequences into amino acids must be specified by the user for tools that perform codon-level operations. EZmito2 supports the following NCBI genetic code tables:

Code	Name
2	The Vertebrate Mitochondrial Code
4	The Mold, Protozoan, and Coelenterate Mitochondrial Code
5	The Invertebrate Mitochondrial Code
9	The Echinoderm and Flatworm Mitochondrial Code
11	The Bacterial, Archaeal and Plant Plastid Code
13	The Ascidian Mitochondrial Code
14	The Alternative Flatworm Mitochondrial Code
21	The Trematode Mitochondrial Code
24	The Rhabdopleuridae Mitochondrial Code
33	The Cephalodiscidae Mitochondrial UAA-Tyr Code

The correct genetic code must be selected before analysis; an incorrect code will cause legitimate codons to be flagged as internal stop codons and the corresponding sequences to be discarded.

2. General Input Requirements

This section defines the input file formats and conventions used across the EZmito2 toolkit. The same conventions apply to all tools, so they are described here once and referenced from the individual tool sections.

2.1 FASTA Format

The majority of EZmito2 tools accept input in FASTA format. The following rules apply universally:

- **Unique identifiers.** Every sequence record within a file must carry a unique identifier (the string following the > character). Duplicate identifiers will cause an error at the validation stage.
- **Consistent taxon names.** When multiple per-gene FASTA files are submitted simultaneously (e.g., to EZpipe, EZcodon, EZskew, or EZtrampo), taxon identifiers must be identical across files. Name mismatches will result in sequences being excluded from concatenation or statistical analyses.
- **IUPAC characters.** Standard nucleotide characters (A, C, G, T) and IUPAC ambiguity codes (R, Y, S, W, K, M, B, D, H, V, N) are accepted. Non-IUPAC characters will trigger a validation error.
- **Internal gaps.** Gap characters (-) are removed automatically from unaligned input files. In turn, tools that require pre-aligned input (EZdist, EZpopstat, EZpcoa), all sequences must have equal length.
- **Internal stop codons.** Sequences containing premature stop codons (i.e., stop codons not at the terminal position) are detected using the selected genetic code table. If premature stop codons are detected, the program halts and the analysis fails. This check is based on the assumption that functional protein-coding genes should not contain internal stop codons.

2.2 Per-gene FASTA Files

Several tools (EZpipe, EZcodon, EZskew, EZtrampo) operate on collections of per-gene FASTA files. The convention is: **one FASTA file per gene**, each containing the unaligned nucleotide sequences of all taxa for that gene. Taxon names must be identical across all gene files (see Section 2.1): a mismatch in any identifier will cause that taxon to be treated as a distinct entry, resulting in an incomplete or incorrect analysis. If a taxon is absent from one or more gene files, the corresponding positions in the concatenated sequence are automatically filled with missing data characters (?).

EZcodon and EZskew additionally require each gene file to be assigned to either the J strand or the N strand (see Section 1.1). EZpipe and EZtrampo do not require strand assignment for the FASTA input itself; EZtrampo can optionally use a gene-order reference to enable strand-aware partitioning.

> **Tip.** Per-gene FASTA files produced by EZsplit (Section 3.1) are directly compatible with EZpipe, EZcodon, EZskew, and EZtrampo. For strand-aware tools (EZcodon, EZskew), the user only needs to sort the EZsplit output files into the J strand and N strand groups according to the species' mitochondrial gene order.

2.3 Annotation Formats

Tools that operate on full annotated mitogenomes (EZcircular, EZmap) accept annotation files in one of two standard formats:

- **BED format (6-column).** Each row defines a genomic feature with the fields: chromosome/sequence name, start coordinate (0-based), end coordinate (exclusive), feature name, score, and strand (`+` or `-`) separated by a `<tab>`.
- **GFF3 format.** Standard General Feature Format version 3 as specified by the Sequence Ontology Consortium, with a mandatory `##gff-version 3` header (further information available at: https://www.ensembl.org/info/website/upload/gff3.html). EZcircular and EZsplit auto-detect the annotation format and convert GFF3 to BED internally when required.

> **Note.** Sequence identifiers in the FASTA file must exactly match the chromosome/sequence names in the annotation file. Any mismatch will prevent feature extraction.

> **Note.** Also EZsplit relies on full annotated mitogenome but strictly requires the companion GFF3 annotation file that is downloaded from NCBI in conjunction with the original genome sequence.

2.4 Population Map and Group Map

Most population-genetic tools (EZpopstat, EZpcoa) optionally accept a tab-separated mapping file that links sequences to populations (EZpopstat, EZpcoa) and populations to higher-level groups (EZpopstat). These files are described here once and referenced from the corresponding tool sections.

Population map (popmap)

A two-column, tab-separated text file with no header. The first column contains the sequence identifier (matching the FASTA header exactly); the second column contains the population label assigned to that sequence. One row per sequence. Sequences absent from the popmap are excluded from population-level statistics. Example:

```
ind001      PopA
ind002      PopA
ind003      PopB
ind004      PopC
```

Group map (groupmap)

A two-column, tab-separated text file with no header. The first column contains the population label (matching the second column of the population map); the second column contains the higher-level group label (e.g., a region, a subspecies, or a clade). One row per population. The group map is only used in conjunction with a population map, and only by tools that perform two-level hierarchical analyses (the hierarchical AMOVA in EZpopstat). Example:

```
PopA  North
PopB  North
PopC  South
```

When a population map is provided without a group map, only one-level statistics (within- *vs* among-population variance) are produced. When both are provided, two-level hierarchical statistics (within-population, among-populations-within-groups, and among-groups variance components) are produced (see Section 3.9).

3. Tool Reference

3.1 EZsplit — Gene Extraction from Annotated Mitogenomes

Purpose

EZsplit automates the extraction of protein-coding gene sequences from annotated mitochondrial genome files available in GenBank (NCBI), producing one FASTA file per gene suitable for downstream alignment and phylogenetic analysis.

Method

The tool reads the genome sequence(s) from the FASTA input and parses features annotated with the attribute `gene_biotype=protein_coding` in the accompanying GFF3 annotation file. For each such feature, the tool:

1. Extracts the nucleotide subsequence defined by the annotation coordinates.
2. Applies reverse-complementation for features on the negative strand (`-`).
3. Matches the extracted gene's name against an internal dictionary of standard mitochondrial gene synonyms, normalising naming inconsistencies (e.g., COX1, COI, cox1 are all recognised as the same gene).
4. Appends the extracted sequence to the corresponding per-gene FASTA file, using the genome record identifier as the sequence name.

A `missing_genes.txt` report lists standard mitochondrial genes that were not found in the annotation. Genes absent from this report are present in all submitted genomes. GFF3 parsing is performed with Biopython's GFF module (https://pypi.org/project/bcbio-gff/).

Inputs and Parameters

Parameter	Format	Required	Description
Genomic FASTA	`.fasta` / `.fa` / `.fna` / `.fas`	Yes	Complete mitogenome(s) sequences
GFF3 annotation	`.gff` / `.gff3`	Yes	Standard GFF3 annotation(s); only protein_coding features are extracted

Outputs

File	Description
`<gene_name>.fasta`	One FASTA file per detected protein-coding gene; each file contains one sequence per input genome
`missing_genes.txt`	List of standard mitochondrial genes absent from the annotation
`log.txt`	Full run log including validation warnings

How To Prepare Input Files

In the NCBI Nucleotide database (https://www.ncbi.nlm.nih.gov/nucleotide/), users can retrieve one or multiple mitochondrial genome records by entering either free-text queries or accession numbers in the search bar. Once the desired records are displayed, both the genome sequence and the annotation can be downloaded by clicking **Send to** → **Complete Record** → **File** → **Format** and selecting FASTA (for the nucleotide sequence) and GFF3 (for the annotation), respectively. The two downloaded files serve as direct input to EZsplit.

Interpreting Outputs

The per-gene FASTA files produced by EZsplit are the primary input for EZpipe (Section 3.4), EZcodon (Section 3.5), EZskew (Section 3.6), and EZtrampo (Section 3.8). Each file should contain one sequence per taxon; if fewer sequences are present than the number of input genomes, the discrepancy is explained in `log.txt` and `missing_genes.txt.` The gene files are named according to the standardised mitochondrial gene nomenclature and should be verified to ensure the correct strand assignment before submission to strand-aware tools.

3.2 EZcircular — Mitogenome Re-orientation

Purpose

EZcircular re-orients a mitochondrial genome sequence and its annotation so that a user-specified gene defines the new coordinate origin. This operation is necessary when comparing mitogenomes with different gene orders, or when a genome downloaded from a public repository uses a non-standard starting point.

Method

For a circular genome, re-orientation is implemented as a circular permutation of the nucleotide sequence. The genome sequence is cut at the start position of the user-specified gene, and the two resulting fragments are swapped and rejoined, so that the chosen gene now begins at position 1. The annotation coordinates are updated accordingly to reflect the new sequence layout. EZcircular can also be used for incomplete (linear) genomes. In this case a stretch of 100 ambiguous nucleotides (Ns) is inserted at the junction point before re-orientation. This artificial gap prevents any annotated feature from being inadvertently split across the new sequence boundary.

The annotation format (BED or GFF3) is detected automatically from the file header, and GFF3 files are converted to BED internally using `pybedtools` (Dale et al. 2011) before processing.

Inputs and Parameters

Parameter	Format	Required	Description
Genomic FASTA	`.fasta` / `.fa` / `.fna` / `.fas`	Yes	Single-sequence mitogenome
Annotation	BED (6-col) or GFF3	Yes	Gene coordinates; format is auto-detected
Topology	circular / linear	Yes	Circular uses modular permutation; linear appends 100 Ns before permutation
Start gene	string	Yes	Standard gene name defining the new coordinate origin (e.g., *COX1*, *trnF*)

Outputs

File	Description
`output.fasta`	Re-oriented genome sequence; the first nucleotide corresponds to the start of the specified gene
`output.bed`	Updated BED annotation with coordinates remapped to the new origin
`log.txt`	Full run log including validation warnings

How To Prepare Input Files

EZcircular input files are intended for use with newly generated mitogenome annotations (e.g., from a MITOS2 run; Bernt et al. 2013) or with data already deposited in publicly available databases (e.g., GenBank). A flexible input format allows users to provide annotations in either GFF3 or BED format alongside the corresponding FASTA sequence. EZcircular is designed to retain biological features (genes, CDS, tRNA, rRNA, D-loop, and related elements) and extracts feature names from the `product`, `gene`, or `Name` attributes, in that order of priority. As such, the starting gene specified by the user must correspond to one of the feature names present in the annotation file; the match is case-insensitive.

Interpreting Outputs

The re-oriented FASTA and BED files are suitable for direct comparison with other mitogenomes sharing the same gene order, or for submission to genome annotation databases.

3.3 EZmap — Mitogenome Graphical Mapping

Purpose

EZmap generates publication-quality graphical representations of mitochondrial genomes directly from annotation files, without requiring a genome sequence. Both circular and linear topologies are supported. Gene features are colour-coded by strand.

Method

Gene coordinates and names are read from the annotation file, which can be supplied as GFF3 or BED format. Each annotated feature is rendered as a proportional arc (circular maps) or rectangle (linear maps) according to its start and end positions relative to the total genome length. Strand assignment is indicated by colour: J strand genes and N strand genes are rendered in distinct user-selectable colours. Feature labels are placed automatically, with overlap resolution to maximise readability. Circular maps are drawn with pyCirclize (https://github.com/moshi4/pyCirclize); linear maps are drawn with Matplotlib (Hunter 2007).

Inputs and Parameters

Parameter	Format / Type	Required	Description
Annotation	BED (6-col) or GFF3	Yes	Genome annotation
Topology	Circular / Linear	Yes	Map shape
J strand colour	Hex colour string	No	Colour for J strand genes
N strand colour	Hex colour string	No	Colour for N strand genes

Outputs

File	Description
`<circular/linear>_plot.pdf`	Circular or linear mitogenome map with proportional gene blocks, strand colours, and gene labels
`log.txt`	Full run log

How To Prepare Input Files

EZmap requires a mitochondrial genome annotation provided in GFF3 or BED format (see Section 2.3). The tool plots all biological features present in the annotation file onto a circular or linear map, including protein-coding genes (CDS), transfer RNA genes (tRNA), ribosomal RNA genes (rRNA), and the control region (D-loop or A+T-rich region). Structural sub-features such as exons, introns, and UTRs are automatically excluded. Feature labels are extracted from the `product`, `gene`, or `Name` attributes of the annotation file, in that order of priority. Genes on the J strand (Heavy strand, encoded on the forward strand) and genes on the N strand (Light strand, encoded on the reverse strand) are rendered in distinct colours selected by the user.

Interpreting Outputs

The mitochondrial genome map provides a visual overview of the gene content, gene order, and strand assignment of the analysed mitogenome. It can be used to verify the completeness of the annotation, to identify missing or duplicated features, and to detect gene order rearrangements relative to a reference species. Arrow orientation indicates strand: arrows pointing clockwise (circular map) or rightward (linear map) represent J strand genes; arrows pointing counterclockwise or leftward represent N strand genes.

3.4 EZpipe — Phylogenetic-ready Nucleotide Matrix

Purpose

EZpipe implements a standardised, fully automated pipeline for preparing multi-gene nucleotide alignments and producing the input files required by partitioned phylogenetic analyses. The tool is designed for large phylo-mitogenomic datasets but is equally applicable to small matrices.

Method

The pipeline executes the following sequential steps:

1. **Input validation.** Each FASTA file is checked for duplicate identifiers, uniform sequence lengths (within-gene), presence of non-IUPAC codes, and internal stop codons (using the selected genetic code).
2. **Gap removal.** Any gap characters are stripped from the nucleotide sequences, yielding ungapped coding sequences.
3. **Translation.** Each gap-free nucleotide sequence is translated to amino acids using the selected NCBI mitochondrial genetic code.
4. **Amino acid alignment.** Amino acid sequences are aligned using MAFFT (Katoh and Standley 2013) with default parameters.
5. **Back-translation.** The amino acid alignment is used to retroalign nucleotide sequences, inserting gap characters at positions corresponding to inserted amino acid residues. This procedure preserves the codon triplet frame throughout the alignment.
6. **Alignment trimming.** Ambiguously aligned blocks are removed using `pyGblocks` (Castresana 2000; iTaxoTools: https://github.com/iTaxoTools/pygblocks) under stringent settings. This step reduces alignment noise that may mislead phylogenetic inference. Fixed parameters employed:

Minimum Number Of Sequences For A Conserved Position	50% of sequences + 1
Minimum Number Of Sequences For A Flank Position	85% of sequences
Maximum Number Of Contiguous Nonconserved Positions	4
Minimum Length Of A Block	10
Allowed Gap Positions	0

7. **Codon position filtering (optional).** If the user selects the two-position option, third codon positions are removed from gene alignments before concatenation. Third positions evolve faster and may saturate over deep evolutionary timescales, potentially introducing noise.
8. **Concatenation and format conversion.** All per-gene alignments are concatenated in the same gene matching taxa by name. The supermatrix is exported in PHYLIP sequential format (`infile.phy`), which is accepted by RAxML, IQ-TREE, and MrBayes.
9. **Partition scheme generation.** A PartitionFinder2 (Lanfear et al. 2017) configuration file is generated specifying codon-based data blocks for each gene. Each gene is subdivided into partitions `p1`, `p2` (and optionally `p3`) corresponding to first, second, (and third) codon positions.

Inputs and Parameters

Parameter	Format / Type	Required	Description
Gene FASTA files	`.fasta` / `.fas` / `.fa` / `.fna`	Yes	Unaligned nucleotide sequences; one file per gene
Genetic code	Integer (see Section 1.2)	Yes	NCBI mitochondrial code for stop-codon validation and translation
Codon positions	2 or 3	Yes	2: retain first and second codon positions only; 3: retain all three positions

Outputs

File	Description
`infile.phy`	Concatenated supermatrix in PHYLIP sequential format; ready for RAxML, IQ-TREE, or MrBayes
`partition_finder.cfg`	PartitionFinder2 configuration file defining codon-position partitions for each gene
`log.txt`	Run log with validation failures and the number of taxa retained per gene

How To Prepare Input Files

EZpipe requires per-gene FASTA files following the convention described in Section 2.2 (one FASTA file per gene, identical taxon names across all files). If a taxon is absent from one or more gene files, the corresponding positions in the final supermatrix are automatically filled with missing data characters (`?`), so that the taxon is retained across all partitions. Files produced by EZsplit (Section 3.1) are directly compatible without further processing. EZpipe and EZtrampo share this identical input convention (the per-gene FASTA files prepared for one tool can be reused as-is for the other).

Interpreting Outputs

The `infile.phy` file is the concatenated supermatrix of retroaligned genes in PHYLIP format, ready for submission to maximum-likelihood or Bayesian phylogenetic inference software. The `partition_finder.cfg` file can be used with PartitionFinder2 to select the best-fit substitution model for each codon-position partition; alternatively, the partition scheme can be adapted for use directly in IQ-TREE (using the `-p`, `-q` or `-Q` option) or MrBayes (using the `partition` command).

The log file should be inspected to identify genes or taxa that were excluded during validation. If sequences are discarded due to internal stop codons, the selected genetic code should be verified, or the original annotation should be reviewed for frameshifts. Genes entirely removed by the Gblocks trimming step are also reported in the log with an explicit warning.

3.5 EZcodon — Codon Usage Analysis

Purpose

EZcodon computes two complementary codon usage statistics: Relative Synonymous Codon Usage (RSCU) and amino acid frequencies; and produces graphical summaries.

Method

EZcodon accepts per-gene FASTA files assigned by the user to either the J strand or the N strand, or both simultaneously. For each strand, all submitted gene files are first validated, degapped, and checked for internal stop codons using the selected genetic code. The sequences are then concatenated into a single per-taxon sequence representing the total coding content of that strand. Ambiguous codons producing ambiguous amino acids are excluded from the analysis with a warning. RSCU values and amino acid frequencies are then computed independently for each taxon on the J strand, the N strand, and, when both strands are provided, the combined JN dataset. RSCU values are computed with the CAI library (Lee 2018), while amino acid frequencies are calculated as the percentage of each amino acid relative to the total number of amino acids in the translated sequence.

Relative Synonymous Codon Usage (RSCU)

Given one amino acid i encoded by n_i synonymous codons (j=1, 2, ..., n_i); assuming that the j-th codon for amino acid i appears X_{ij} times in the genome; $RSCU_{ij}$, i.e. the relative synonymous codon usage of codon j for amino acid i, is defined as (Sharp and Li 1986):

$$RSCU_{ij} = \frac{X_{ij} n_i}{\sum_{j=1}^{n_i} X_{ij}}$$

An RSCU value of 1.0 indicates that the codon is used exactly as often as expected under uniform synonymous usage. Values greater than 1.0 indicate over-representation; values below 1.0 indicate under-representation. Stop codons and codons producing ambiguous amino acids are excluded from the calculation.

Amino Acid Frequency

The relative frequency of each amino acid is computed as the number of occurrences of that residue divided by the total number of residues in the translated concatenated sequence for each taxon, expressed as a percentage.

Inputs and Parameters

Parameter	Format	Required	Description
J strand gene files	`.fasta` / `.fas` / `.fa` / `.fna`	At least one strand	Per-gene FASTA files for Heavy-strand genes
N strand gene files	`.fasta` / `.fas` / `.fa` / `.fna`	At least one strand	Per-gene FASTA files for Light-strand genes
Genetic code	Integer	Yes	NCBI mitochondrial code

Outputs

File	Description
`<strand>_RSCU.csv`	Comma-separated table of RSCU values per codon and taxon and strand (J, N, or JN)
`<strand>_AAfreq.csv`	Comma-separated table of amino acid frequencies per taxon and strand (J, N, or JN)
`<strand>_RSCU.pdf`	Stacked bar chart showing RSCU per synonymous codon group and taxon
`<strand>_Aminoacid_frequency.pdf`	Line plot (≤20 taxa) or box plot (>20 taxa) of amino acid frequencies across taxa
`log.txt`	Full run log

How To Prepare Input Files

EZcodon requires per-gene FASTA files following the convention described in Section 2.2, with the additional requirement that each file be assigned by the user to either the J strand or the N strand (see Section 1.1). Files produced by EZsplit (Section 3.1) are directly compatible: the user only needs to sort the EZsplit output files into the J strand and N strand groups according to the species' mitochondrial gene order. EZskew (Section 3.6) uses an identical input convention, so the same set of files can be reused for both tools without modification.

Interpreting Outputs

In the RSCU plots, each bar represents a synonymous codon group, stacked by the individual synonymous codons contributing to it. Bars with uniform proportions across taxa indicate homogeneous codon preferences; variable proportions indicate inter-taxon differences in codon usage. In the amino acid frequency plots, taxa that deviate markedly from the overall distribution may indicate misidentified sequences, contamination, or genuine biological divergence in protein composition.

3.6 EZskew — Nucleotide Compositional Bias Analysis

Purpose

EZskew quantifies and visualises nucleotide compositional biases across the protein-coding gene complement of mitochondrial genomes at three levels: overall nucleotide content, strand-specific composition, and codon-position-specific skew. These biases reflect the asymmetric mutational pressures experienced by the two mitochondrial strands during replication and transcription. During mitochondrial replication in metazoans, the two strands spend asymmetric time in the single-stranded state. The displaced J strand (Heavy strand) remains single-stranded for a longer period and is therefore subject to deamination of cytosine to uracil (C→T transitions) and oxidative damage to guanine (G→T transversions). This produces a strand-specific excess of A+C on the J strand and G+T on the N strand, measurable as AC% and GT% respectively, and detectable as asymmetric AT-skew and CG-skew values when computed separately for each codon position (Lobry 1996; Perna and Kocher 1995; Hassanin et al. 2005). Because the third codon position evolves under weaker amino acid constraints than the first and second positions, it accumulates strand bias most rapidly and provides the clearest signal of mutational pressure.

Method

EZskew accepts per-gene FASTA files assigned by the user to either the J strand or the N strand, or both simultaneously. For each strand, all submitted gene files are first validated, degapped, and checked for internal stop codons using the selected genetic code. Sequences are padded to a uniform length and concatenated into a single per-taxon sequence representing the total coding content of that strand. Three compositional metrics are then computed following Hassanin et al. (2005):

- **AT%** (strand-independent) is computed over the full concatenated protein-coding gene matrix and reflects the overall adenine+thymine content of the mitochondrial coding sequences.
- **AC%** is computed over the J strand concatenated matrix only and reflects the adenine+cytosine content of the Heavy strand genes.
- **GT%** is computed over the N strand concatenated matrix only and reflects the guanine+thymine content of the Light strand genes.

These three values are plotted together as a grouped bar chart per taxon. When more than 30 taxa are analysed, an additional frequency histogram is produced to summarise the distribution of AT%, AC%, and GT% values across the dataset.

AT-skew and CG-skew are then computed separately for the 1st, 2nd, and 3rd codon positions over the J and N strand concatenated matrices, using the formulas:

$$ATskew = \frac{A - T}{A + T}$$

$$CGskew = \frac{C - G}{C + G}$$

Both statistics range between −1 and +1. Results are reported per taxon and per strand, and plotted as a three-panel scatter plot (one panel per codon position) in which J strand values are shown as circles and N strand values as triangles. When more than 20 taxa are analysed, the species colour legend is suppressed automatically to preserve readability. Nucleotide content is computed with Biopython (Cock et al. 2009).

Inputs and Parameters

Parameter	Format	Required	Description
J strand gene files	`.fasta` / `.fas` / `.fa` / `.fna`	At least one strand	Per-gene FASTA files for J strand genes
N strand gene files	`.fasta` / `.fas` / `.fa` / `.fna`	At least one strand	Per-gene FASTA files for N strand genes
Genetic code	Integer	Yes	NCBI mitochondrial code

Outputs

File	Description
`Final_table.tsv`	Tab-separated file with all the values per taxon
`First_and_second_bias.pdf`	Bar-plot of AT% strand independent bias, AC% J strand bias, GT% N strand bias per taxon
`Third_bias.pdf`	Three-panel scatter plot of AT-skew vs CG-skew at the 1st, 2nd, and 3rd codon positions per taxon per strand
`log.txt`	Full run log

How To Prepare Input Files

Input preparation for EZskew is identical to that of EZcodon (Section 3.5): per-gene FASTA files (see Section 2.2) assigned by the user to the J strand or the N strand. The same set of files can be reused without modification for both tools. Files produced by EZsplit (Section 3.1) are directly compatible after strand assignment.

Interpreting Outputs

Metazoan mitogenomes typically show a pronounced positive AT-skew on the J strand (more A than T) and a negative CG-skew (more C than G), consistent with strand-asymmetric deamination. The strand bias plot allows direct comparison between the two strands: if both strands show the same skew direction, the signal is likely driven by selection rather than replication asymmetry. The codon-position plots are particularly diagnostic because 3rd-position skew is unconfounded by amino acid constraints and reflects neutral mutational pressure most directly. Deviations from the expected pattern (e.g., a reversal of CG-skew) may indicate the presence of an alternative replication origin or compositional adaptation to extreme environments.

3.7 EZmix — Inter-mitogenome Similarity Detection

Purpose

EZmix identifies regions of high nucleotide similarity between pairs of candidate mitogenome sequences. Its primary application is the detection of chimeric assembly artefacts (i.e., sequences that erroneously incorporate fragments from two or more distinct biological molecules) but it is also applicable to the study of inter-specific or inter-individual sequence sharing in mitogenomic contexts. Chimeric assemblies can arise when a pool of related mitogenomes is sequenced as a single library, for example to reduce library costs or following a metagenomic approach, without physical sample barcoding, and assembled *de novo* (Cucini et al. 2021) to the extent that some mixing can occur. Chimeric mitogenomes can introduce severe topological errors in phylogenetic analyses because they combine evolutionary signals from different biological sources. Detection of chimeras prior to phylogenetic analysis is therefore a critical quality-control step.

Method

EZmix performs pairwise all-against-all BLASTn comparisons (Camacho et al. 2009; `blastn -task blastn`) among all submitted sequences. Prior to comparison, input sequences are validated and gap characters are removed. Each unique sequence pair is then compared sequentially: for each pair, one sequence is used as query and the other as subject in all orientations, and the BLAST output is parsed to retain only hits satisfying two user-defined thresholds:

- **Minimum identity (%).** Only hits with a pairwise nucleotide identity at or above this threshold are retained.
- **Minimum length (bp).** Only hits spanning at least this number of nucleotides are retained.

The results are rendered in a linear diagram in which each genome sequence is drawn as a thin horizontal line proportional to its length. Similarity regions detected between a pair of genomes are highlighted as thick black segments on both the query and subject lines, and a coloured diagonal line connecting the midpoints of the two corresponding segments is drawn, visually linking the similar regions across the two genomes. Each sequence pair is assigned a distinct colour, so that all similarity regions shared between any two sequences are immediately identifiable across the plot.

Inputs and Parameters

Parameter	Format / Type	Required	Description
Multi-sequence mitogenome	`.fasta` / `.fas` / `.fa` / `.fna`	Yes	Two or more complete or partial mitogenome sequences; sequences are compared in all pairwise combinations
Minimum identity	50–100%	Yes	Percentage identity threshold below which similarity regions are discarded
Minimum length	Integer (bp)	Yes	Length threshold below which similarity regions are discarded

Outputs

File	Description
`plot.pdf`	Linear diagram of all sequences with similarity (mixed) regions displayed
`log.txt`	Full run log

How To Prepare Input Files

EZmix accepts a multi-FASTA file containing two or more mitochondrial genomes suspected of inter-sequence chimeric assembly. The identity and length thresholds are customisable. By default, both are set to conservative values in order to detect only unambiguous chimeric misassemblies. However, optimal thresholds strictly depend on the taxon, sequencing approach, and assembly method, so no universal rule can be easily defined. Lowering either threshold will increase the number of detected similarity regions.

Interpreting Outputs

Similarity regions found at unexpected genomic positions are strong candidates for chimeric assembly. By contrast, genuine biological conservation, such as conserved rRNA-coding regions or shared haplotypes among closely related taxa, produces similarity concentrated at homologous positions across sequences. All sequences flagged by EZmix should be verified by mapping the original sequencing reads back to the assembly prior to inclusion in phylogenetic analyses. If no similarity regions exceeding the specified thresholds are detected, the output plot will show only bare lines representing genomes with no connecting segments, indicating the absence of detectable inter-sequence mixing under the chosen parameters.

3.8 EZtrampo — Transmembrane Domain Partition Analysis

Purpose

EZtrampo is a Python implementation of the TRAMPO (TRAnsMembrane Protein Order) pipeline (Cucini et al. 2026), designed to generate phylogenetic partition schemes based on the secondary protein structure of mitochondrial inner-membrane proteins. By annotating each codon with its transmembrane domain assignment, EZtrampo produces a suite of NEXUS partition files that can be directly used as data block definitions in Bayesian or maximum-likelihood phylogenetic software.

The thirteen mitochondrial protein-coding genes encode subunits of the oxidative phosphorylation complexes, several of which are integral membrane proteins with transmembrane (TM) helical segments spanning the inner mitochondrial membrane. These segments are embedded in a hydrophobic lipid environment and experience markedly different selective pressures from the hydrophilic regions facing the mitochondrial matrix (MA) or the inter-membrane space (IM). This domain-specific selection produces distinct nucleotide compositional signatures at each codon position within TM, MA, and IM regions (Cucini et al. 2026). Ignoring this heterogeneity in phylogenetic analyses may lead to substitution model mis-specification. EZtrampo provides partition files that allow phylogenetic inference software to fit independent substitution models to each domain–codon position combination.

Method

EZtrampo executes the following sequential steps:

1. **Input validation.** Gene FASTA files are renamed to match standard mitochondrial gene nomenclature, then validated for duplicate identifiers, sequence length outliers, IUPAC compliance, and internal stop codons using the selected genetic code. If fewer than 13 gene files are submitted, a warning is recorded in the log. Gap characters are removed before further processing.
2. **Translation and amino acid alignment.** Each validated nucleotide sequence is translated to amino acids using the selected genetic code. Amino acid sequences are aligned with MAFFT (Katoh and Standley, 2013).
3. **Model organism integration.** The reference amino acid sequence of the chosen model organism is added to each per-gene amino acid alignment using MAFFT `--add`. The alignment is then back-translated to nucleotides, preserving the codon triplet frame throughout.
4. **Domain partitioning.** Each per-gene amino acid alignment is sliced into TM, MA, and IM charsets based on an internal curated database of the model organism. Domain labels assigned at the amino acid level are projected back to the corresponding nucleotide triplets.
5. **Partition scheme generation.** Charset coordinates across all genes are extracted, offset to their concatenated supermatrix positions, and combined into NEXUS partition files (see **Outputs** subsection). NEXUS output is written via Biopython's Nexus module (Cock et al. 2009). Mann–Whitney tests on per-domain compositional metrics are computed with SciPy (Virtanen et al. 2020), with multiple-testing correction provided by statsmodels (Seabold and Perktold 2010).

Available Model Organisms

Nickname	Species	Taxon
hsa	*Homo sapiens*	Mammalia
lse	*Lycodon semicarinatus*	Lepidosauria
gga	*Gallus gallus*	Aves
xle	*Xenopus laevis*	Amphibia
dre	*Danio rerio*	Actinopterygii
sca	*Scyliorhinus canicula*	Chondrichthyes
pma	*Petromyzon marinus*	Agnatha
ppe	*Patiria pectinifera*	Asteroidea
spu	*Strongylocentrotus purpuratus*	Echinoidea
dme	*Drosophila melanogaster*	Pancrustacea
rsa	*Rhipicephalus sanguineus*	Chelicerata
aca	*Albinaria caerulea*	Mollusca
lte	*Lumbricus terrestris*	Annelida
cel	*Caenorhabditis elegans* (*ATP8* absent; will be skipped)	Nematoda
mse	*Metridium senile*	Cnidaria
user	Custom model	—

Available Gene Order References (strand-aware partitioning)

Nickname	Taxon
vert	All vertebrates
panc	Arthropods
ances	*L. terrestris*, *C. elegans*, *M. senile*
albin	*A. caerulea*

Inputs and Parameters

Parameter	Format / Type	Required	Description
Gene FASTA files	`.fasta` / `.fas` / `.fa` / `.fna`	Yes	Unaligned nucleotide sequences; one file per gene
Genetic code	Integer	Yes	NCBI mitochondrial code
Model organism	Selection from list	Yes	Reference organism for internal database correspondence; select `user` to provide a custom TMHMM 2.0 (Krogh et al. 2001) table prediction
Reference AA sequence	`.fasta` / `.fas` / `.fa` / `.fna`	If model = `user`	Custom reference protein sequence in amino acid format (see TRAMPO documentation for header format)
TMHMM tables	One `.txt` per gene	If model = `user`	TMHMM 2.0 output files generated externally with the TMHMM software
Gene order reference	Selection or –	No	Enables strand-aware partition schemes; set to `-` to disable strand partitioning

Outputs

EZtrampo writes its output across multiple folders and a collection of NEXUS partition files, summarised separately below.

Output Files and Folders

File / Folder	Description
`nt_combined.nex`	Concatenated nucleotide supermatrix in NEXUS format
`partitions/`	Partition files in NEXUS format (see below)
`tables/`	TSV tables of amino acid frequencies, RSCU values, GC content, and AT/CG skew per domain
`plots/`	HTML and PDF graphical outputs per domain (and per strand if gene order is provided)
`stats/`	Summary statistics and Mann–Whitney test results
`log.txt`	Full run log including validation warnings and runtime

NEXUS Partition Files

NEXUS file	Partitions	Partition basis
`3p_cod.nex`	3	Codon position (1st, 2nd, 3rd)
`3p_dom.nex`	3	Protein domain (TM, MA, IM)
`9p_dom_cod.nex`	9	Domain × codon position
`13p_gen.nex`	13 (one per gene)	Gene
`39p_gen_dom.nex`	39	Gene × domain
`117p_gen_dom_cod.nex`	117	Gene × domain × codon position
`6p_cod_str.nex`	6	Codon position × strand *(requires gene order)*
`6p_str_dom.nex`	6	Strand × domain *(requires gene order)*
`12p_MAIM_merged.nex`	12	Codon position × domain (MA+IM merged) *(requires gene order)*
`18p_str_dom_cod.nex`	18	Strand × domain × codon position *(requires gene order)*

How To Prepare Input Files

The basic FASTA input requirement for EZtrampo is identical to that of EZpipe (Section 3.4): one FASTA file per gene, with consistent taxon names across all files (see Section 2.2). The same per-gene FASTA collection can be reused without modification across the two tools. Files produced by EZsplit (Section 3.1) are directly compatible.

EZtrampo additionally expects the thirteen mitochondrial protein-coding genes to be present, and filenames must follow accepted gene name conventions; a full list of accepted synonyms is given in the table below. When fewer than 13 gene files are provided, a warning is issued but the analysis proceeds with the available genes.

If the `user` model is selected, a custom amino acid FASTA file and a folder of TMHMM 2.0 table files (one per gene) must be provided. Gene names must appear in the FASTA headers following the accepted naming convention. TMHMM 2.0 (Krogh et al. 2001) must be run externally on the reference organism and the resulting table files organised into a dedicated folder before submission to EZtrampo.

Gene	Accepted filenames (case-insensitive)
ATP6	ATP6, A6, MT-ATP6, ATPASE6
ATP8	ATP8, A8, MT-ATP8, ATPASE8
COX1	COX1, CO1, COXI, MT-CO1, MTCO1, MTCOX1, MTCOXI
COX2	COX2, CO2, COXII, MT-CO2, MTCO2, MTCOX2, MTCOXII
COX3	COX3, CO3, COXIII, MT-CO3, MTCO3, MTCOX3, MTCOXIII
CYTB	CYTB, COB, MT-CYB, MTCYB
ND1	ND1, NAD1, NADH1, MT-ND1, MTND1
ND2	ND2, NAD2, NADH2, MT-ND2, MTND2
ND3	ND3, NAD3, NADH3, MT-ND3, MTND3
ND4	ND4, NAD4, NADH4, MT-ND4, MTND4
ND4L	NDL, NAD4L, NADH4L, MT-ND4L, MTND4L
ND5	ND5, NAD5, NADH5, MT-ND5, MTND5
ND6	ND6, NAD6, NADH6, MT-ND6, MTND6

Interpreting Outputs

The choice of partition scheme should be guided by model selection criteria (AIC, BIC) computed by PartitionFinder2 or IQ-TREE. Simpler schemes (e.g., `3p_cod.nex`) are computationally efficient and suitable for exploratory analyses. The most detailed scheme (`117p_gen_dom_cod.nex`) captures the maximum biological heterogeneity but requires substantially more computational resources. Strand-aware partition files are only produced when a gene order reference is specified and should be preferred when the dataset spans taxa with a conserved gene order.

The domain-specific RSCU and amino acid frequency plots in the `plots/` folder provide a diagnostic visualisation of compositional differences between TM, MA, and IM regions, and can be used to verify that the topology prediction was correctly applied to the query sequences. Mann–Whitney test results in the `stats/` folder report whether nucleotide compositional metrics differ significantly between domains.

3.9 EZpopstat — Population Genetics Statistics

Purpose

EZpopstat computes standard population genetics statistics from an aligned mitochondrial sequence dataset. It is designed for intraspecific or shallow interspecific datasets where population structure is the primary object of study (e.g an haplotype alignment) and each sequence represents an individual.

Method

EZpopstat operates on a pre-aligned multi-FASTA dataset. When a population map is provided (see Section 2.4), sequences are assigned to populations and per-population and pairwise statistics are computed; when a group map is also provided, two-level hierarchical statistics are computed in addition. Pairwise allele-frequency operations are delegated to scikit-allel (https://github.com/cggh/scikit-allel); permutation-based p-values are computed by random label shuffling for the requested replicate count. Haplotype collapsing produces a unique-haplotype FASTA file with frequency and population annotations in the header following the FABox convention, suitable for direct use in haplotype network construction (e.g., TCS; Clement et al. 2000; PopART; Leigh et al. 2015) or as input to EZpcoa.

Per-population statistics

- n: sample size (number of sequences).
- h: number of distinct haplotypes. Two sequences are considered the same haplotype if and only if they are identical at all sites after pairwise complete deletion of ambiguous positions.
- H (haplotype diversity): the probability that two randomly selected sequences belong to different haplotypes as implemented in scikit-allel (`haplotype_diversity`; https://scikit-allel.readthedocs.io)
- S (segregating sites): the number of nucleotide positions that are variable across at least two sequences within the population, computed after per-population complete deletion of sites with ambiguous characters.
- π (nucleotide diversity): the average proportion of sites that differ between randomly chosen pairs of as implemented in scikit-allel (`sequence_diversity`; https://scikit-allel.readthedocs.io).
- Tajima's D: a test statistic comparing two estimators of the population mutation rate (Tajima, 1989): one based on the number of segregating sites S, and one based on π. Under the standard neutral model with constant population size, Tajima's D is computed following Tajima (1989) as implemented in scikit-allel (`tajima_d`; https://scikit-allel.readthedocs.io). Significantly negative values indicate an excess of rare variants relative to neutral expectations, consistent with a recent selective sweep or population expansion; significantly positive values indicate an excess of intermediate-frequency variants, consistent with balancing selection or population contraction.

Pairwise differentiation

When a population map is provided, EZpopstat estimates pairwise F_{ST} between all population pairs using the Hudson (1992) estimator as elaborated by Bhatia et al. (2013) and implemented in scikit-allel (`hudson_fst`; https://scikit-allel.readthedocs.io). For each pair of populations, per-site mean pairwise differences are computed within each population and between populations, and F_{ST} is estimated as a ratio of sums across all segregating sites. Note that values may be negative when within-population diversity exceeds between-population divergence.

Analysis of molecular variance (AMOVA)

AMOVA (Excoffier et al. 1992) partitions the total molecular variance into hierarchical components. EZpopstat uses raw Hamming distances and global complete deletion (only sites without ambiguous characters in any sequence are used). Two models are available:

- One-level AMOVA (population map only). Partitions variance into within-population and among-population components. The Φ_{ST} statistic is the fraction of total variance attributable to differences among populations.
- Two-level hierarchical AMOVA (population map + group map). Partitions variance into within-population (Φ_{ST}), among populations within groups (Φ_{SC}), and among groups (Φ_{CT}) components. The three Φ statistics sum to explain the total variance.

P-values are estimated by label permutation: the population (or group) labels are randomly shuffled a user-specified number of times (0, 999, or 9999), and the proportion of permuted statistics as extreme as the observed value is used as the p-value. 9999 permutations are recommended for final reporting.

Inputs and Parameters

Parameter	Format	Required	Description
Aligned FASTA	`.fasta` / `.fas` / `.fa` / `.fna`	Yes	Pre-aligned sequences; all must have equal length
Population map	Tab-separated TSV	No	Format described in Section 2.4. Sequences absent from the map are excluded from population-level statistics.
Group map	Tab-separated TSV	No (requires population map)	Format described in Section 2.4. Enables two-level hierarchical AMOVA.
Permutations	0 / 999 / 9999	Yes	Number of permutations for AMOVA p-value estimation

Outputs

File	Description
population_statistics.tsv	Per-population values of n, h, H (±SD), S, π (±SD), and Tajima's D
haplotype_assignments.tsv	Mapping of each sequence identifier to its haplotype ID
collapsed_haplotypes.fasta	Unique haplotypes with frequency and population annotations in the header
FST_pairwise.tsv	Pairwise Hudson FST estimates between all population pairs (if population map provided)
AMOVA_summary.tsv	AMOVA results: variance components, Φ statistics, and permutation p-values
log.txt	Run log

How To Prepare Input Files

EZpopstat requires a single multi-FASTA file containing pre-aligned mitochondrial sequences (e.g., a *cytb* or *cox1* alignment from a population sample). All sequences must have equal length after alignment, and ambiguous IUPAC characters are handled by pairwise or global complete deletion as specified in the Method subsection.

Optionally, a population map and a group map can be supplied to enable population-level and hierarchical statistics. Both files follow the format described in Section 2.4. Sequence identifiers in the population map must match the FASTA headers exactly.

Interpreting Outputs

Per-population values of haplotype diversity (H), nucleotide diversity (π), and Tajima's D should be interpreted jointly. Low H and low π typically indicate recent bottlenecks or founder effects; high values of both indicate large effective population size or population admixture. Significantly negative Tajima's D supports a recent selective sweep or demographic expansion, whereas significantly positive values are consistent with balancing selection or recent population contraction.

Pairwise F_{ST} values approaching 1 (rarely above 0.5 on real data) indicate strong genetic differentiation between population pairs (limited gene flow or long isolation), whereas values close to 0 indicate panmixia. The AMOVA summary file reports how the total molecular variance is partitioned across hierarchical levels: a substantial Φ_{CT} indicates that the supplied grouping captures real biological structure, while a near-zero Φ_{CT} indicates that the proposed grouping does not explain genetic variation. P-values should be reported alongside the corresponding Φ statistics.

3.10 EZpcoa — Ordination of Genetic Variation

Purpose

EZpcoa performs ordination analysis on aligned DNA sequences to summarise and visualise patterns of genetic variation in a reduced-dimensional space. Unlike neighbour-joining or maximum-likelihood trees, ordination methods do not assume a hierarchical (bifurcating) structure.

Method

Ordination method is implemented with the scientific Python packages (NumPy, SciPy and scikit-learn; Pedregosa et al. 2011) while plots are produced with Matplotlib (Hunter 2007).

Principal Coordinates Analysis (PCoA)

PCoA (also known as classical Multidimensional Scaling, MDS) operates on a pairwise genetic distance matrix. After collapsing identical haplotypes, EZpcoa computes either the *p*-distance or the Kimura two-parameter (K2P) distance between all pairs of sequences. The squared distance matrix is then double-centered (Gower 1966) and eigendecomposed via NumPy. The resulting principal coordinate axes provide a Euclidean representation in which between-point distances approximate the original genetic distances; each eigenvalue divided by the sum of all positive eigenvalues gives the proportion of total variance explained by that axis. Ambiguous IUPAC characters are skipped pairwise.

The *p*-distance is the simplest genetic distance measure, defined as the proportion of sites at which two sequences differ. It does not correct for multiple hits and is therefore most appropriate for closely related sequences with low divergence and is calculated via scikit-bio (`pdist`; Anton et al. 2026).

$$p = \frac{Number\ of\ differing\ sites}{Total\ number\ of\ sites}$$

The K2P distance model (Kimura 1980) corrects for multiple hits at the same site by assuming two classes of substitution: transitions (P) and transversions (Q), and is computed via scikit-bio (`k2p`; Anton et al. 2026):

$$D = \frac{-1}{2} \ln\left[(1-2P-Q)\cdot\sqrt{1-2Q}\right]$$

Haplotype Visualisation

Unique haplotypes are identified and their ordination coordinates are computed as the mean PC scores of all sequences sharing that haplotype. Each haplotype is rendered as a pie chart at its ordination position: the radius of the pie is proportional to the square root of haplotype frequency (so that the visible area scales linearly with frequency), and slices are coloured by population. Shared haplotypes (i.e., those occurring in more than one population) are visible as multi-coloured pies.

Inputs and Parameters

Parameter	Format / Type	Required	Description
Aligned FASTA	`.fasta` / `.fa` / `.fna` / `.fsa`	Yes	Pre-aligned sequences
Population map	Tab-separated TSV	No	Same format as EZpopstat (see Section 2.4); without a map all sequences are treated as one population
Distance model	`p` / `k2p`	Yes	*p*-distance (uncorrected) or Kimura two-parameter distance
Number of components	Integer (default 3)	Yes	Number of PC axes to compute and export

Outputs

File	Description
`<PCOA>_PC*.pdf`	Pie plot of each haplotype scaled by frequency
`<PCOA>_screeplot.pdf`	Bar chart of eigenvalues showing the proportion of variance explained by each axis
`PC_coordinates.tsv`	PC scores per sequence and per principal coordinate axis
`eigenvalues.tsv`	Eigenvalues and cumulative % variance explained per axis
`distance_matrix.tsv`	Pairwise genetic distance matrix
`log.txt`	Full run log

How To Prepare Input Files

EZpcoa accepts a single multi-FASTA file of pre-aligned sequences. The same alignment used as input to EZpopstat (Section 3.9) and EZdist (Section 3.11) can be reused as-is. A population map (Section 2.4) is optional but recommended: without a population map, all sequences are treated as belonging to a single population and the pie-chart colouring will be uniform.

Interpreting Outputs

The ordination plot should be read in conjunction with the scree plot: if the first two axes explain >60–70% of the total variance, the 2D representation captures most of the genetic structure. Tightly clustered sequences from the same population indicate low intra-population diversity; well-separated clusters indicate divergent lineages. Haplotypes shared across populations (multi-coloured pies) suggest either gene flow, incomplete lineage sorting, or sampling of the same lineage in different locations. Outlier points (single sequences far from their population cluster) should be inspected for possible contamination or misassignment or genuine biological variation.

3.11 EZdist — Ordination of Genetic Variation

Purpose

EZdist computes pairwise genetic distances between all sequences in an aligned FASTA file and produces distance matrices and publication-quality heatmaps. It simultaneously collapses identical sequences into unique haplotypes, allowing users to assess genetic differentiation both at the sequence and haplotype levels. The tool is designed for diversity assessment studies.

Method

EZdist computes pairwise genetic distances between all sequences in an aligned FASTA file using either *p*-distance (uncorrected) or Kimura two-parameter (K2P) distance (see section 3.10 for details). Gap characters and ambiguous IUPAC nucleotides are handled according to the user's gap treatment preference: pairwise deletion (distances computed ignoring gap positions on a pair-by-pair basis) or complete deletion (columns containing gaps in any sequence are removed globally before analysis).

Identical sequences are identified and collapsed into unique haplotypes, each being assigned a label and a frequency count. Two distance matrices are produced: one for the unique haplotypes only (collapsed view) and one for all input sequences (full view).

Distance matrices are rendered as heatmaps ordered by hierarchical clustering (average linkage), with colour intensity proportional to the pairwise distance. Heatmap rows and columns are arranged so that similar sequences cluster together, revealing the structure of genetic variation in the dataset.

Inputs and Parameters

Parameter	Format / Type	Require d	Description
Aligned FASTA	`.fasta` / `.fa` / `.fna` / `.fsa`	Yes	Pre-aligned sequences
Distance model	`p` / `k2p`	Yes	*p*-distance (uncorrected) or Kimura two-parameter distance
Gap treatment	pairwise / complete	Yes	pairwise: skip gaps per pair; complete: remove columns with any gaps
Show values	Boolean	No	If enabled distance values are overlaid on heatmap cells

Outputs

File	Description
`K2P_heatmap_collapsed.pdf`	Heatmap of pairwise distances of unique haplotypes only
`K2P_heatmap_all_sequences.pdf`	Heatmap of pairwise distances of unique all input sequences
`K2P_distance_matrix.tsv`	Tab-separated format of pairwise distance matrix
`haplotype_assignments.tsv`	Mapping of each sequence identifier to its haplotype ID
`collapsed_haplotypes.fasta`	Unique haplotypes with frequency and population annotations in the header
`log.txt`	Full run log

How To Prepare Input Files

EZdist accepts a single multi-FASTA file of pre-aligned sequences. The same alignment used as input to EZpopstat (Section 3.9) and EZpcoa (Section 3.10) can be reused as-is.

Interpreting Outputs

The distance matrix reports pairwise distances expressed as percentages; values close to zero indicate nearly identical sequences, while larger values indicate greater genetic divergence. The matrix can be inspected to identify sequence pairs with unexpectedly large distances, which may indicate contamination, misidentification, or the presence of divergent lineages within the dataset. The collapsed heatmap provides a synthetic overview of the genetic structure of the dataset at the haplotype level, removing the visual noise introduced by redundant identical sequences. In both heatmaps, sequences are re-ordered by hierarchical clustering so that closely related sequences appear adjacent; visually distinct blocks of low-distance cells correspond to genetically cohesive groups.

4. Running EZmito2 Locally

EZmito2 is also distributed as a single Python script (`ezmito.py`) and uses a standard subcommand interface. The general call pattern is:

```
python ezmito.py <tool> [options]
```

Tool-specific help is displayed with the command:

```
python ezmito.py <tool> --help
```

The subsections below list each tool's flags; required arguments are marked with an asterisk (*).

4.1 EZsplit

```
python ezmito.py ezsplit -i genomes.fasta -g annotation.gff3 [-o outdir/]
```

*`-i / --input` Multi-FASTA of complete mitogenomes.

*`-g / --gff` GFF3 annotation file (NCBI format).

`-o / --outdir` Output directory [default: outdir/].

4.2 EZcircular

```
python ezmito.py ezcircular -i genome.fasta -b annotation.gff3 [-s cox1] [-f circular] [-o outdir/]
```

*`-i / --input` Single-sequence mitogenome FASTA.

*`-b / --bed` Annotation file in BED (6-column) or GFF3 format; auto-detected and converted if needed.

`-s / --start` Gene name for new coordinate origin [default: cox1].

`-f / --feature` Genome topology: circular or linear [default: circular].

4.3 EZmap

```
python ezmito.py ezmap -g annotation.gff3 [-f circular] [-colJ “#add8e6”] [-colN “#B22222”] [-o outdir/]
```

*`-g / --gff`	Annotation file in GFF3 or BED format.
`-f / --feature`	Map shape: circular or linear [default: circular].
`-colJ / --colorJ`	Hex colour for J-strand genes [default: #add8e6].
`-colN / --colorN`	Hex colour for N-strand genes [default: #B22222].

4.4 EZpipe

```
python ezmito.py ezpipe -i genes/ -c 2 [-p 3] [-o outdir/]
```

*`-i / --input`	Directory of per-gene FASTA files.
*`-c / --code`	NCBI genetic code (see Section 1.2).
`-p / --positions`	Codon positions to retain: 2 (remove 3rd) or 3 (keep all) [default: 3].

4.5 EZcodon

```
python ezmito.py ezcodon [-J heavy/] [-N light/] -c 2 [-o outdir/]
```

At least one of `-J` or `-N` is required.

`-J / --heavy`	Directory of J strand FASTA files.
`-N / --light`	Directory of N strand FASTA files.
*`-c / --code`	NCBI genetic code.

4.6 EZskew

```
python ezmito.py ezskew [-J heavy/] [-N light/] -c 2 [-o outdir/]
```

At least one of `-J` or `-N` is required.

`-J / --heavy` J strand FASTA directory.

`-N / --light` N strand FASTA directory.

*`-c / --code` NCBI genetic code.

4.7 EZmix

```
python ezmito.py ezmix -i assemblies.fasta [-id 0.90] [-len 500] [-bn
/path/to/blast/] [-o outdir/]
```

*`-i / --input` Multi-FASTA of sequences to screen for chimerism.

`-id / --identity` Minimum fractional identity, 0.5–1 [default: 0.90].

`-len / --length` Minimum hit length in bp [default: 500].

`-bn / --blastn` Path to directory containing the blastn executable [default: system PATH].

4.8 EZtrampo

```
python ezmito.py eztrampo -p genes/ -c 2 -m hsa [-g vert] [-n 4] [-s ref.fasta
-t tables/] [-o outdir/]
```

*`-p / --path` Directory of per-gene FASTA files (up to 13 PCGs).

*`-c / --code` NCBI genetic code.

*`-m / --model` Model organism nickname (e.g., hsa, dme, aca; see Section 3.8).

`-g / --gene_order` Gene order reference for strand-aware partitions (e.g., vert, panc; omit to disable).

`-n / --threads` MAFFT threads [default: 1].

`-s / --sequence` Custom model amino acid FASTA (requires `-m user` and `-t`).

`-t / --tables` Directory of TMHMM 2.0 table files (requires `-m user` and `-s`).

4.9 EZpopstat

```
python ezmito.py ezpopstat -i alignment.fasta [--popmap popmap.txt] [--groupmap groupmap.txt] [--n_perms 999] [-o outdir/]
```

`*-i / --input`	Pre-aligned FASTA.
`--popmap`	Tab-separated population map (see Section 2.4).
`--groupmap`	Tab-separated group map; enables two-level AMOVA (see Section 2.4).
`--n_perms`	AMOVA permutations: 0 (skip) 999 (fast), or 9999 (precise) [default: 9999].

4.10 EZpcoa

```
python ezmito.py ezpcoa -i alignment.fasta [--popmap popmap.txt] [--dist_model k2p] [-n 3] [-p Set1] [-o outdir/]
```

`*-i / --input`	Pre-aligned FASTA.
`--popmap`	Population map (see Section 2.4).
`--dist_model`	Distance model: p or k2p [default: k2p].
`-n / --n_components`	Number of principal coordinates [default: 3].
`-p / --palette`	Matplotlib colour palette for populations [default: Set1].

4.11 EZdist

```
python ezmito.py ezdist -i alignment.fasta [-m k2p] [-g pairwise] [-p Blues] [--show_values] [-o outdir/]
```

`*-i / --input`	Pre-aligned FASTA.
`-m / --model`	Distance model: p or k2p [default: p].
`-g / --gap_treatment`	Gap handling: pairwise or complete [default: pairwise].
`-p / --palette`	Heatmap colour palette [default: Blues].
`--show_values`	Overlay numeric distances on heatmap cells (flag, no value).

5. References

Aton M, McDonald D, Cañardo Alastuey J, Azom R, Batra P, Bezshapkin V, Bolyen E, Cagle A, Caporaso JG, Debelius JW, et al. 2026. Scikit-bio: a fundamental Python library for biological omic data analysis. *Nat Methods.* 23:274-276.

Bernt M, Donath A, Jühling F, Externbrink F, Florentz C, Fritzsch G, Pütz J, Middendorf M, Stadler PF. 2013. MITOS: improved *de novo* metazoan mitochondrial genome annotation. *Mol Phylogenet Evol.* 69:313–319.

Bhatia G, Patterson N, Sankararaman S, Price AL. 2013. Estimating and interpreting FST: the impact of rare variants. *Genome Res*. 23:1514–1521.

Camacho C, Coulouris G, Avagyan V, Ma N, Papadopoulos J, Bealer K, Madden TL. 2009. BLAST+: architecture and applications. *BMC Bioinform.* 10:421

Castresana J. 2000. Selection of conserved blocks from multiple alignments for their use in phylogenetic analysis. *Mol Biol Evol.* 17:540–552.

Clement M, Posada D, Crandall KA. 2000. TCS: a computer program to estimate gene genealogies. *Mol Ecol.* 9:1657-1660.

Cock PJA, Antao T, Chang JT, Chapman BA, Cox CJ, Dalke A, Friedberg I, Hamelryck T, Kauff F, Wilczynski B, et al. 2009. Biopython: freely available Python tools for computational molecular biology and bioinformatics. *Bioinformatics.* 25:1422–1423

Cucini C, Carapelli A, Brunetti C, Molero-Baltanás R, Gaju-Ricart M, Nardi F. 2021. Characterization of the complete mitochondrial genome of *Neoasterolepisma foreli* (Insecta: Zygentoma: Lepismatidae) and the phylogeny of basal Ectognatha. *Mitochondrial DNA B Resour*. 6:119–121.

Cucini C, Nardi F, Pons J. 2026. Integrating secondary structure information enhances phylogenetic signal in mitochondrial protein coding genes. *Syst. Biol. Syag027.*

Dale RK, Pedersen BS, Quinlan AR. 2011. Pybedtools: a flexible Python library for manipulating genomic datasets and annotations. *Bioinformatics*. 27:3423–3424

Excoffier L, Smouse PE, Quattro JM. 1992. Analysis of molecular variance inferred from metric distances among DNA haplotypes: application to human mitochondrial DNA restriction data. *Genetics*. 131:479–491.

Gower JC. 1966. Some distance properties of latent root and vector methods used in multivariate analysis. *Biometrika.* 53:325–338.

Harris CR, Millman KJ, Van Der Walt SJ, Gommers R, Virtanen P, Cournapeau D, Wieser E, Taylor J, Berg S, Smith NJ, et al. 2020. Array programming with NumPy. *Nature.* 585:357–362.

Hassanin A, Léger N, Deutsch J. 2005. Evidence for Multiple Reversals of Asymmetric Mutational Constraints during the Evolution of the Mitochondrial Genome of Metazoa, and Consequences for Phylogenetic Inferences. *Syst. Biol.* 54:277–298.

Hudson RR. 1992. Estimation of levels of gene flow from DNA sequence data. *Genetics.* 132:583–589.

Hunter JD. 2007. Matplotlib: a 2D graphics environment. *Comput. Sci. Eng.* 9:90–95.

Katoh K, Standley DM. 2013. MAFFT multiple sequence alignment software version 7: improvements in performance and usability. *Mol Biol Evol.* 30:772–780.

Kimura M. 1980. A simple method for estimating evolutionary rates of base substitutions through comparative studies of nucleotide sequences. *J Mol Evol.* 16:111–120.

Krogh A, Larsson B, Von Heijne G, Sonnhammer ELL. 2001. Predicting transmembrane protein topology with a hidden markov model: application to complete genomes. *J Mol Biol*. 305:567–580.

Lanfear R, Frandsen PB, Wright AM, Senfeld T, Calcott B. 2016. PartitionFinder 2: New methods for selecting partitioned models of evolution for molecular and morphological phylogenetic analyses. *Mol Biol Evol*. 34:772-773

Lee BD. 2018. Python implementation of codon adaptation index. *J Open Source Softw.* 3:905.

Leigh JW, Bryant D, Nakagawa S. 2015. POPART: full-feature software for haplotype network construction. *Methods Ecol Evol.* 6:1110-1116.

Lobry JR. 1996. Asymmetric substitution patterns in the two DNA strands of bacteria. *Mol Biol Evol.* 13:660–665.

McKinney W. 2010. Data structures for statistical computing in Python. Proceedings of the 9th Python in Science Conference. 56–61.

Pedregosa, F, Varoquaux, G, Gramfort, A, Michel, V, Thirion, B, Grisel, O, Blondel, M, Prettenhofer, P, Weiss, R, Dubourg, V, et al. 2011. Scikit-learn: machine learning in Python. *J Mach Learn Res.* 12:2825–2830.

Perna NT, Kocher TD. 1995. Patterns of nucleotide composition at fourfold degenerate sites of animal mitochondrial genomes. *J Mol Evol.* 41:353–358.

Plotly Technologies Inc. 2015. Collaborative data science. Plotly Technologies Inc., Montréal. https://plot.ly.

Seabold S, Perktold J. 2010. statsmodels: econometric and statistical modeling with Python. In: Proceedings of the 9th Python in Science Conference, 92–96.

Sharp PM, Li WH. 1986. An evolutionary perspective on synonymous codon usage in unicellular organisms. *J Mol Evol.* 24:28–38.

Tajima F. 1989. Statistical method for testing the neutral mutation hypothesis by DNA polymorphism. *Genetics.* 123:585–595.

Virtanen P, Gommers R, Oliphant TE, Haberland M, Reddy T, Cournapeau D, Burovski E, Peterson P, Weckesser W, Bright J, et al. (2020). SciPy 1.0: fundamental algorithms for scientific computing in Python. *Nat Methods.* 17:261–272.
